# BODE index and depression in COPD patients: A cross-sectional study from central India

**DOI:** 10.6026/973206300221511

**Published:** 2026-03-31

**Authors:** Satyendra Mishra, Mahendra Singh Chouhan, Sourabh Jain, Sanjay Gupta

**Affiliations:** 1 Department of Respiratory Medicine, Bundelkhand Medical College, Sagar, Madhya Pradesh, India; 2 Department of Pediatrics, Bundelkhand Medical College, Sagar, Madhya Pradesh, India; 3 Department of Emergency Medicine, Bundelkhand Medical College, Sagar, Madhya Pradesh, India; 4 Department of TB and Respiratory Diseases, Chief Medical Officer (Senior Administrative Grade), NITRD, Delhi, India

**Keywords:** Chronic Obstructive Pulmonary Disease (COPD), depression, body mass index, obstruction, Dyspnea and Exercise capacity (BODE) index, Patient Health Questionnaire-9 (PHQ-9)

## Abstract

Depression is a common comorbidity in Chronic Obstructive Pulmonary Disease (COPD) patients, exacerbating their disease burden. The BODE index, which assesses COPD severity, includes parameters such as Body mass index, obstruction, dyspnea and exercise capacity. Therefore, it is of interest to explore the correlation between the BODE index and depression in 160 clinically stable COPD patients. A significant positive correlation was found between the BODE index and depression severity (ρ=0.44, P<0.01). This study advances knowledge by highlighting the link between the BODE index and depression, emphasizing the need for integrated care approaches.

## Background:

Chronic Obstructive Pulmonary Disease (COPD) is a major global health problem, ranked as the third leading cause of death, responsible for 3.23 million deaths in 2019 [1^1^]. The burden is disproportionately higher in middle and low- income countries. COPD is characterized by a progressive decline in respiratory function, leading to significant physical limitations and chronic symptoms like dyspnea and cough. The prevalence of psychiatric disorders, particularly depression, is notably higher in COPD patients compared to the general population [2]. Depression adversely affects the quality of life and increases the frequency of acute exacerbations in COPD patients, yet it remains under diagnosed and undertreated [3]. Several models, including the cognitive-behavioural model and the carbon dioxide hypersensitivity model, have been proposed to explain the pathophysiological link between COPD and depression. Persistent dyspnea in COPD patients is thought to stimulate the amygdala and limbic system, contributing to chronic depression [4]. Studies report a varying prevalence of depression in COPD patients, ranging from 30% to 90%, highlighting the need for further research into associated risk factors [5]. Therefore, it is of interest to investigate the correlation between the BODE index and depression in COPD patients, providing insights into the impact of disease severity on mental health.

## Methodology:

This cross-sectional study was conducted at the Government Bundelkhand Medical College, Sagar, India. Clinically stable COPD patients, diagnosed according to GOLD guidelines and attending the respiratory outpatient department, were recruited. Patients with significant comorbid conditions, such as uncontrolled hypertension, ischemic heart disease, active tuberculosis, severe pulmonary hypertension, hepatic dysfunction, metastatic cancer, renal failure, cognitive deficits and psychiatric diseases interfering with compliance, were excluded. HIV-positive and pregnant patients were also excluded. Ethical approval was obtained from the institutional research committee. Patients were briefed about the study and provided informed consent. Clinical history, including COPD duration, hospitalizations, ICU admissions, smoking history, education, occupation, residence, family income and family type, was collected. Physical examinations and routine investigations, including spirometry, were performed.

## Depression:

Assess using the Patient Health Questionnaire-9 (PHQ-9), a validated tool for diagnosing and measuring depression severity [6].

##  BODE index:

Calculated using body mass index (BMI), forced expiratory volume in 1 second (FEV1), Modified Medical Research Council (mMRC) dyspnea scale and 6-minute walk test (6MWT) (7) Data were analyzed using SPSS software. The correlation between the BODE index and depression was evaluated using Spearman's correlation coefficient, with statistical significance set at P<0.05.

## Results:

Depression was significantly more prevalent among male patients and smokers. Increasing age showed a higher proportion of depression; however, the association was not statistically significant. A statistically significant positive correlation was observed between BODE index scores and depression severity. The prevalence of depression increased progressively with rising BODE index categories. More than one-fourth of patients had mild depressive symptoms, while moderate to severe depression was observed in approximately 19% of the study population. Low BMI, severe airflow limitation, higher dyspnea grades and reduced exercise capacity were independently associated with a higher prevalence of depression. Depression prevalence increased significantly with advancing GOLD stage, indicating a strong association between disease severity and psychological morbidity. Depression was significantly more prevalent among male patients and smokers. Increasing age showed a higher proportion of depression, but this association was not statistically significant. A statistically significant positive correlation was observed between BODE index scores and depression severity; with the prevalence of depression increasing progressively with rising BODE index categories. More than one-fourth of patients exhibited mild depressive symptoms, while moderate to severe depression was observed in approximately 19% of the study population.

Low BMI, severe airflow limitation, higher dyspnea grades, and reduced exercise capacity were independently associated with a higher prevalence of depression. Depression prevalence increased significantly with advancing GOLD stage, indicating a strong association between disease severity and psychological morbidity. Table 1 (see PDF) shows the association of demographic variables with depression in COPD patients. Male patients exhibited a significantly higher prevalence of depression (50.81%) compared to females (28.57%) (p = 0.02). Smokers also had a significantly higher prevalence of depression (45.87%) compared to non-smokers (41.18%) (p < 0.01). Table 2 (see PDF) presents the distribution of depression according to BODE index categories. Depression prevalence increased with higher BODE index categories, with the highest prevalence observed in the 7-10 BODE category (85.71%) (p < 0.01). Table 3 (see PDF) details the distribution of depression severity according to the PHQ-9 score. The majority of patients (55.62%) had no depression, followed by those with mild depression (25.62%) and moderate depression (11.25%). Fewer patients had moderately severe depression (5.63%) or severe depression (1.88%). Table 4 (see PDF) highlights the association of individual BODE components with depression. Low BMI (<21 kg/m^2^) was significantly associated with higher depression prevalence (60.87%) (p < 0.01). Severe airflow limitation (FEV1 <50%) was also significantly associated with depression (61.63%) (p < 0.01). Higher dyspnea grades (≥2) and shorter 6-minute walk distances (<350 m) were associated with higher depression prevalence, both with p-values less than 0.01. Table 5 (see PDF) shows the relationship between GOLD stage and depression prevalence. Depression prevalence increased significantly with advancing GOLD stage, with the highest prevalence in Stage IV (61.76%) (p < 0.01).

## Discussion:

The present study highlights a strong association between the severity of Chronic Obstructive Pulmonary Disease (COPD) and the prevalence as well as severity of depression. Nearly 44.38% of the study population was found to have depressive symptoms, emphasizing depression as a frequent and clinically relevant comorbidity in COPD patients. This finding is consistent with previous studies reporting a high burden of depression among individuals with COPD [6]. Analysis of demographic variables revealed that depression was more prevalent among male patients and smokers. Smoking has been shown to worsen respiratory symptoms, accelerate disease progression and contribute to systemic inflammation, all of which may predispose patients to depressive symptoms [7]. Although a higher proportion of depression was observed in patients aged 60 years and above, the association was not statistically significant. Similar observations have been reported in earlier studies, suggesting that factors other than age alone, such as disease severity and functional limitation, may play a more dominant role in determining mental health outcomes in COPD [8]. According to the severity based on PHQ-9 scores showed that mild depression was the most common presentation, while nearly one-fifth of patients had moderate to severe depression. Even mild depressive symptoms can adversely affect physical activity levels, treatment adherence and overall quality of life in COPD patients, underscoring the importance of early detection and intervention [9]. A key finding of the present study is the significant positive correlation between the BODE index and depression severity. Patients with higher BODE index scores demonstrated a markedly increased prevalence of depression, with the highest rates observed in those with scores between 7 and 10. BODE index, by incorporating body mass index, airflow obstruction, dyspnea severity and exercise capacity, provides a multidimensional assessment of disease severity. This comprehensive nature makes it particularly relevant for predicting psychological outcomes compared to spirometric parameters alone [10]. Further analysis of individual components of the BODE index revealed that low BMI, severe airflow limitation, higher dyspnea grades and reduced six-minute walk distance were all significantly associated with depression. Among these, dyspnea severity and reduced exercise capacity showed the strongest association. Persistent breathlessness can induce fear, anxiety, activity avoidance and social isolation, which may progressively lead to depressive symptoms. These findings support earlier studies that identified dyspnea and functional impairment as major contributors to depression in COPD patients [11, 12]. In addition, a progressive increase in depression prevalence was observed with advancing GOLD stages. Patients in GOLD stage III and IV exhibited significantly higher rates of depression compared to those in early stages of the disease. This observation reinforces the concept that worsening disease severity and functional decline are closely linked to psychological morbidity in COPD [13]. Overall, the findings of this study emphasize the need for an integrated and holistic approach to COPD management. Routine screening for depression using validated tools such as PHQ-9 should be incorporated into standard clinical practice, particularly for patients with higher BODE index scores, advanced GOLD stages and significant functional limitations. Early identification and management of depression may improve treatment adherence, reduce exacerbation frequency and enhance overall quality of life in COPD patients [14].

## Conclusion:

Higher BODE index scores are significantly associated with an increased prevalence and severity of depression in patients with COPD. Depression was more common in patients with greater functional impairment and advanced disease stages. Routine screening for depression and an integrated approach addressing both physical and psychological aspects of COPD are essential to improve overall patient outcomes and quality of life.
